# RoboEM: automated 3D flight tracing for synaptic-resolution connectomics

**DOI:** 10.1038/s41592-024-02226-5

**Published:** 2024-03-21

**Authors:** Martin Schmidt, Alessandro Motta, Meike Sievers, Moritz Helmstaedter

**Affiliations:** 1 Department of Connectomics, Max Planck Institute for Brain Research, Frankfurt, Germany; 2 Faculty of Science, Radboud University, Nijmegen, the Netherlands

**Keywords:** Computational neuroscience, Synaptic transmission, Mouse

## Abstract

Mapping neuronal networks from three-dimensional electron microscopy (3D-EM) data still poses substantial reconstruction challenges, in particular for thin axons. Currently available automated image segmentation methods require manual proofreading for many types of connectomic analysis. Here we introduce RoboEM, an artificial intelligence-based self-steering 3D ‘flight’ system trained to navigate along neurites using only 3D-EM data as input. Applied to 3D-EM data from mouse and human cortex, RoboEM substantially improves automated state-of-the-art segmentations and can replace manual proofreading for more complex connectomic analysis problems, yielding computational annotation cost for cortical connectomes about 400-fold lower than the cost of manual error correction.

## Abstract (web summary)

RoboEM enables automated proofreading of electron microscopy datasets using a strategy akin to that of self-steering cars. This decreases the need for manual proofreading of segmented datasets and facilitates connectomic analyses.

## Main

**Fig. 1:**
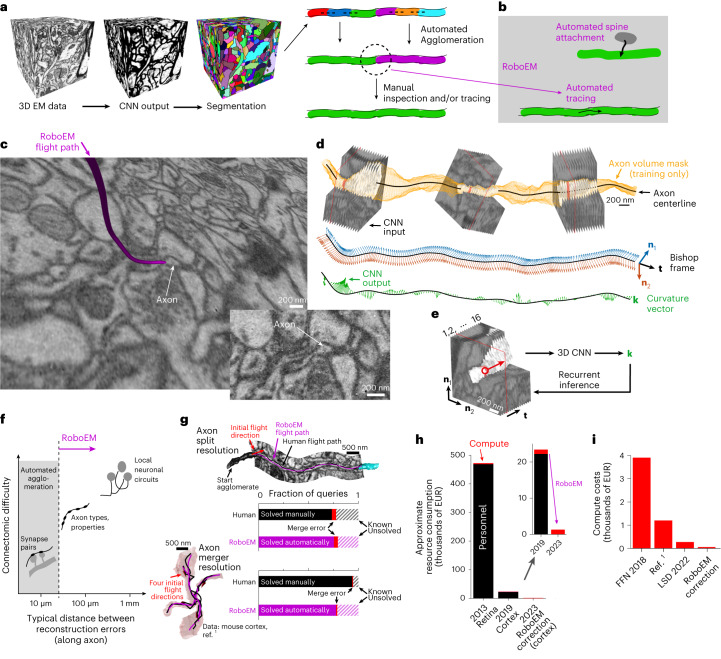
Automated neurite tracing for substitution of human annotation needs in neuronal network reconstruction. **a**, Standard workflow for connectomic analyses from 3D-EM data: initial volume segmentation (two-step process of classification and watershed^17,22,24–28^, or directly as foreground classification^30–32^); automated agglomeration (based on interfaces between segments); manual inspection to resolve remaining errors and reach reconstruction quality usable for meaningful connectomic analysis^1,2,12,16,19,37–41^. Data cubes with 10 µm edge lengths are shown. **b**, RoboEM replaces human inspection and correction step by automated connection and/or validation flights solving split and merge errors and attaching remaining spine heads^1^. **c**, Example of the RoboEM flight path along a thin axon in SBEM data^1^. **d**, Design of RoboEM: volumetric EM data as input for prediction of a steering vector that determines the subsequent input. Yellow denotes segmentation mask for ‘teaching’ corrective steering signals from off-center locations (during training, only). **e**, Detailed sketch of RoboEM inference setup. **f**, Calibration of reconstruction automation by the difficulty of automatable connectomic analyses comes from synaptic pairs-based analyses^1,12,37^ via extraction of axonal properties^1,21^ (Fig. 2b and Supplementary Fig. 1) to local neuronal circuits. **g**, RoboEM performance in direct comparison to human annotators on axon ending (*n* = 90) and chiasma queries (*n* = 100) for split and merge error resolution^1^. **h**, Effect on resource consumption for connectomic dense reconstructions. **i**, Computing costs for state-of-the-art segmentation and agglomeration: FFNs^32^ and local shape descriptors (LSD)^25^ are compared against a dense connectomic reconstruction^1^ (additionally including costs for synapse and type predictions and processing of human and/or RoboEM skeletons). EUR, euros. Source data

Extracting the dense neuronal connectivity from three-dimensional electron microscopy (3D-EM) data of brain tissues poses major computational challenges^1–6^. Substantial progress in the field has allowed us to move from fully manual skeleton reconstructions of neurites^3,6–11^ via combinations of skeleton reconstruction and automated segmentations^5,12^ to proofreading of automated image segmentation (Fig. 1a). This proofreading was initially as laborious as fully manual skeleton reconstructions^4,13–15^ but has recently been made more efficient by focused human intervention based on improved automated segmentations^1,2,12,16^. Yet, even for automated methods claiming super-human performance^17^ or full automation^12^, when applied to large-scale EM datasets, massive manual-annotation efforts are required for the intended connectomic analyses^2,18–20^.

Connectomic analyses differ widely in their reconstruction difficulty: for the analysis of pairs of neighboring synapses along a neurite for extraction of learned synaptic configurations^1,12^, for example, axon reconstructions of about 10 µm in length are sufficient, and therefore these analyses can already now be fully automated^1,12^. Obtaining neuron-to-neuron connectomes from cortical tissue, however, requires faithful axon reconstruction for at least an additional order of magnitude of axonal length, and has so far not been possible fully automatically. Intermediate-scale connectomic analyses, aimed at axonal synaptic properties for example, demand error-free axonal reconstruction in the range of 30–50 µm (corresponding to about 4–10 synapses per axon stretch, depending on species and tolerable error rates of the intended analysis^1,21^) (Supplementary Note and Supplementary Fig. 1). Such error-free lengths are not yet fully automatically accessible.

Connectomic image analyses have in common that EM data are processed by artificial neural networks to yield voxel-based maps reporting plasma membranes^17,22–28^, or the similarity between pairs of image voxels^29^, or the association of image voxels to the same foreground object^30–32^. Then, segmentations are computed, and automated methods for the joining or splitting of these initial segmentation objects are currently the main focus of computational improvements^17,24,30–36^. When automated approaches become insufficient, human annotation is used to solve the most challenging of these joining or splitting operations, ideally directed to difficult locations by computational means^1,2,12,37–41^, or by human inspection^16,19^. The FocusEM^1^ toolset is one such focused reconstruction approach, which automatically detects reconstruction errors and asks human annotators to fly along axons^42^ until another segmentation object is reached and thus the problematic location resolved (Fig. 1b). Notably, the usage of image sequences along neurites for alleviating manual reconstruction was already considered in the early days of EM-based neurite tracing^43–47^. More recently, iterative contour tracking methods have been proposed to automate the neurite-following process^48–51^, even if these were not yet applied at scale and not learned. Here, by contrast, we wondered whether an artificial neural network could learn to directly control the steering along neurites in 3D and in an end-to-end fashion.

## Results

**Extended Data Fig. 1:**
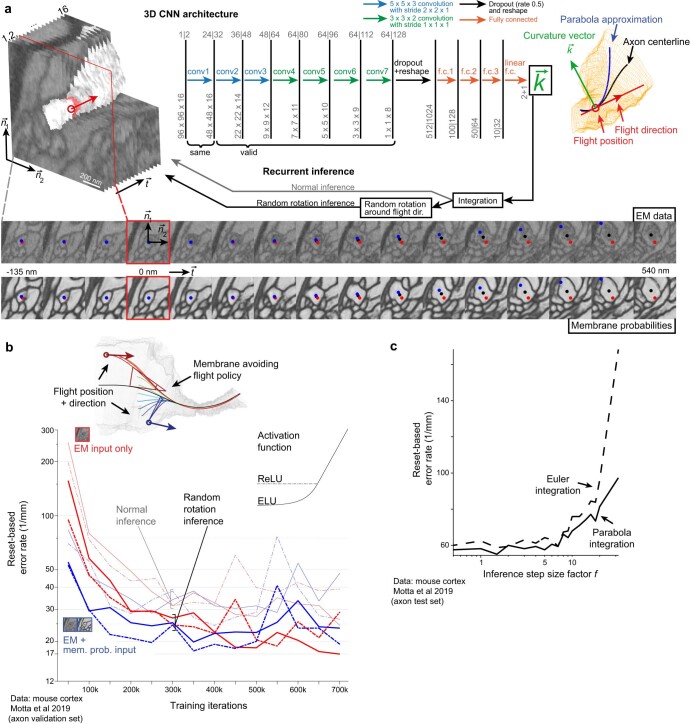
Automated flight reconstruction. **(a)** 3D Convolutional neural network architecture variants processing projections of neurite-aligned subvolumes of EM input data and making predictions about neurite continuation in terms of a curvature vector; EM data from Motta et al.^1^. This curvature vector is representative of a parabola approximation to the neurite centerline and can be integrated during recurrent inference to yield the next position and orientation and thereby the next subvolume to be processed. Performing random rotations around the flight direction during inference can be considered zero-cost test time augmentation that allows to decorrelate subsequent inputs with respect to the orientation. The sequence of visited points during recurrent inference represents a skeleton reconstruction of the neurite. **(b)** Training iterations using a membrane avoiding flight policy for off-center and off-direction inputs and comparing ReLU and ELU activation functions^66,67^, as well as EM only input versus EM and membrane probability maps as input. EM data was used for predicting neurite continuation. Random rotation inference mode as depicted in (a) was used. **(c)** Reset-based evaluation of RoboEM for different step size factors f on the test set axons from the mouse cortext dataset^1^. (f = 1.5). Source data

The process of flight tracing along elongated (often very thin) neurites via steering commands has an analogy to image-based road following in autonomous driving^52,53^. Based on this analogy, we developed a convolutional neural network (CNN) architecture to output 3D steering signals directly from neurite-aligned 3D-EM image volumes. We (1) defined a continuous 3D steering framework; (2) defined a membrane-avoiding flight policy to recover from off-centerline positions and noncenterline-aligned orientations; (3) trained a CNN on image-steering pairs from on- and off-centerline positions and orientations in a supervised manner allowing for stable path following during recurrent inference; and (4) defined a validation strategy for automated error detection (Fig. 1c–e and Extended Data Fig. 1a,b).

In particular, we used the Bishop frame^54^ along interpolated neurite centerline reconstructions to describe the neurites’ local directionality, defined the projection for the neurite-aligned 3D-EM input and then used the corresponding Bishop curvatures as the target output steering signals that had to be predicted (Fig. 1d). During training only, a neurite volume mask (obtained by segment pick-up from an oversegmentation) was used to generate off-centerline inputs and corresponding target output steering signals back toward the centerline (Extended Data Fig. 1b). During inference, predicted Bishop curvatures were integrated to yield the next position and orientation (Extended Data Fig. 1c). This approach yielded an automated neurite path annotation agent that mimicked the process of human flight-mode annotation in 3D (ref. ^42^) (Supplementary Video 1), called RoboEM.

Next, we investigated to what degree RoboEM could in fact replace human annotation, and which kinds of connectomic analysis would thus become fully automatable (Fig. 1f). For this, we started with a set of dense connectomic analyses of a piece of mammalian neocortex in which, based on automated segmentations, human annotators had been asked to resolve an automatically identified set of problem locations consuming a total of 4,000 work hours^1^. To resolve split errors, endings of axons and spine necks had been queried, asking the human annotators to continue if possible until the task was automatically stopped when another reconstructed object of sufficient size had been reached (Fig. 1g). To resolve merger errors, chiasmatic configurations of axons had been detected and queried, asking the human annotators for proper continuation from one chiasmatic exit into one of the other exits (Fig. 1g). We used RoboEM to replace these human annotations. For this, we issued RoboEM queries analogous to human annotator queries and required that, additionally, a forward continuation along a neurite should be confirmed by tracing the same neurite location backward (automatic validation). We found that 76% of the ending queries and 78% of the chiasma queries were traced and validated by RoboEM without errors (as judged by manual inspection, compared to fully manual queries yielding 74 and 94%, respectively). Restricting to RoboEM annotations in which forward and backward tracings agreed allowed avoiding most merge errors, yielding only 4% of queries with RoboEM-introduced tracing errors for ending tasks and 1% for chiasma tasks, similar to human annotations.

While this performance indicated that RoboEM could accurately replace human annotation for a range of connectomic analyses, we wanted to quantify this conclusion explicitly by using connectomic analysis itself as the metric for reconstruction success. As described above, some connectomic analyses require more reconstruction accuracy than others. In particular, we considered: A1, paired same-axon same-dendrite synapse analyses aimed at measuring the learned fraction of a connectome^1^; A2, spine rate analyses for identification of interneuron dendrites and A3, axonal type analysis based on the synaptic target distribution of axons. We then used three types of connectome for comparison: CI, the connectome obtained from the fully automated reconstruction, before any focused human annotation^1^; CII, the connectome including 4,000 work hours of human annotation^1^ and CIII, the connectome obtained from combining the fully automated reconstruction CI with RoboEM, yielding a fully automated and automatically proofread connectome. We then performed the three types of connectomic analysis, which we expected to increase in connectomic difficulty.

**Extended Data Fig. 2:**
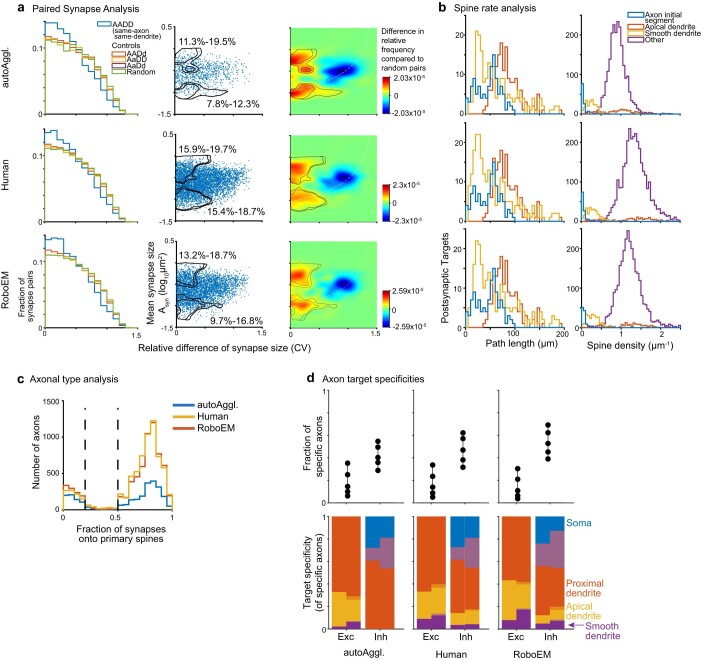
Connectomic analyses compared across reconstruction states. Analyses based on EM data and reconstructions from Motta et al.^1^ **(a)** Paired same-axon same-dendrite analysis yielding similar fractions of paired synapses that are consistent with Hebbian learning. **(b)** Spine rate analysis yielding underestimates of apical dendrite spine densities for the reconstruction state before RoboEM or manual-annotation-based spine attachment. **(c)** Connectomic definition of excitatory and inhibitory axons with at least 10 synapses based on fraction of primary spine innervation, compared for the automated state (AutoAggl.) before RoboEM, the manual-annotation-based split and merge resolution of axons, and RoboEM application. **(d)** Axonal target specificities of excitatory and inhibitory axons.

When applying the analyses A1–A3 to the three stages of connectomes CI–CIII, we found that the analysis of paired synapses for quantifying potentially learned synapses in the connectome A1 was already possible with the automated connectome state before any RoboEM-based corrections (CI, upper bound of fraction of paired connections consistent with long term potentiation: CI 11–20%; CII 16–20%; CIII 13–19%, Extended Data Fig. 2a). However, for obtaining correct spine rates for apical dendrites (A2, CI 0.9 ± 0.4; CII 1.3 ± 0.6; CIII 1.2 ± 0.5 spines per µm, mean ± s.d., Extended Data Fig. 2b) and the true fraction of excitatory axons defined by their spine head preference (excitatory axon fractions, A3, CI 75%; CII 87%; CIII 84%, Extended Data Fig. 2c), manual or RoboEM-based corrections were required. In addition, RoboEM-based corrections recovered the axonal target specificities of inhibitory axons onto apical dendrites and smooth dendrites, whereas in the automated state before any annotation this specificity was not detectable (A3, one-sided Kolmogorov–Smirnov test, CI apical dendrites *P* = 1.0%, smooth dendrites *P* = 3.6%; CII apical dendrites *P* = 2.7 × 10^−4^, smooth dendrites *P* = 1.8 × 10^−3^ and CIII apical dendrites *P* = 1.9 × 10^−5^, smooth dendrites *P* = 7.1 × 10^−4^, Extended Data Fig. 2d). Thus, using the difficulty of connectomic analyses that can be fully automated as the criterion for evaluating RoboEM-based error correction, we found a shift of automated analysis performance from simpler to more complex connectomic problems (Fig. 1f), yielding 400-fold reduced annotation costs compared to manual error correction (Fig. 1h). The computational cost was 5- to 80-fold lower than other approaches (Fig. 1i and Supplementary Table 4), rendering RoboEM a suitable candidate to run automated error correction as a postprocessing step. These results were obtained on 3D-EM data imaged with serial block-face scanning electron microscopy (SBEM)^55^.

**Fig. 2:**
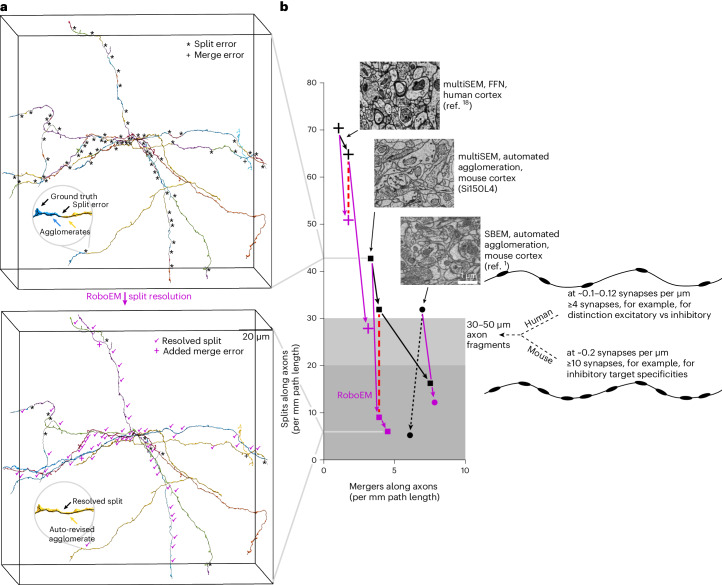
RoboEM improves state-of-the-art connectomic reconstruction results in mammalian cortex. **a**, Example of dense axon sets used for unbiased calibration of reconstruction success. Agglomerates before (top) and after RoboEM correction (bottom). **b**, Quantification of axon split and merge error rate for randomly seeded axons, evaluated on various 3D-EM data: human cortex multiSEM dataset^18^ (crosses) (two agglomeration states analyzed (c2,c3) with 8% split resolution from c3 to c2); mouse cortex multiSEM dataset (Si150L4, https://wklink.org/7122); mouse cortex SBEM dataset^1^. Purple symbols show the effect of applying RoboEM to previous reconstruction. The red dashed line denotes the same merge-rate comparison for human data (3.5-fold improved split resolution) and mouse data (3.1-fold improved split resolution). Purple lines denote RoboEM split-rate improvement with modest merge rate increases yielding error rates tolerable for more complex connectomic analyses (Fig. 1f). The black dashed line denotes a human annotator-based error resolution (as published in ref. ^1^) shown for comparison. Source data

Next, we applied RoboEM to data obtained using a state-of-the-art high-throughput 3D-EM imaging approach for mm^3^-scale volumes from mammalian brains^18,21^ (ATUM-multiSEM, where ATUM stands for automated tape-collecting ultramicrotome^56^ followed by multibeam scanning electron microscope (multiSEM)^57^). We used a (150 µm)^3^ subvolume centered on cortical layer 4, subsequently referred to as ‘Si150L4’, cropped from a larger 1.3 × 1.3 × 0.25 mm^3^ sized 3D-EM dataset that had been imaged at 4 × 4 × 35 nm^3^ voxel size spanning all cortical layers of mouse primary somatosensory cortex (S1). In this subvolume, we quantified RoboEM performance on axons sampled uniformly from the volume (‘densely seeded axons’, Fig. 2a,b). After automated agglomeration, we seeded RoboEM at automatically detected endings of axon agglomerates, and used it to connect possible missing axonal agglomerates (Fig. 2a). As a result, split rates of axons were reduced sevenfold (42.7 to 6.0 per mm axon path length) while only modestly increasing merger rates (3.3 to 4.5 per mm, Fig. 2b).

To titrate the effect of RoboEM separately for split and merge errors, we used the fact that automated agglomerations (before RoboEM application) typically have a split-versus-merger parameter that can be adjusted independently. We then introduced a similar parameter for RoboEM, and could therefore directly compare the split rates achieved by automated agglomeration and RoboEM at equal merge error rates. We found (Fig. 2b) that the agglomeration solved 25% of split errors yielding 31.9 splits per mm, while RoboEM solved 79% yielding 9.0 splits per mm, compared at 3.9 merge errors per mm axon path length. Hence, in this split and/or merge error regime RoboEM improves split resolution 3.1-fold over state-of-the-art agglomeration (Fig. 2b). The resulting axon reconstructions were comparable to those achieved in the local cortical SBEM dataset^1^ (5.2 split and 6.1 merge errors per mm, Fig. 2b).

Similar to thin axons, thin spine necks can pose difficulties for automated reconstruction pipelines of 3D-EM data. To assign synapses onto spine heads to the correct postsynaptic neuron, spine necks need to be reconstructed with high accuracy. We used RoboEM to follow automatically detected spine heads along the spine neck back to the dendritic shaft of origin. Evaluated on a subvolume (Si11L3) in cortical layer 3 sized (11 µm)^3^ from the above ATUM-multiSEM dataset of mouse S1 cortex, we found that spine head attachment recall increased from 70 to 94%, while retaining high precision of 97% (85 out of 91 test spine heads attached to the correct dendrite, 3 out of 91 spine heads attached to the wrong dendrite and 3 out of 91 spine heads were unattached, of which one could also not be attached manually and was therefore counted as true negative).

Finally, we wondered whether RoboEM could also be applied for improving existing state-of-the-art automated segmentations that are currently being manually proofread and had been generated by flood-filling networks (FFN)^18,32^. We evaluated RoboEM on a subvolume of size (150 µm)^3^ from a recently published mm^3^-scale ATUM-multiSEM dataset from human cortex^18^ and found that application of RoboEM on axonal endings obtained from the dense reconstructions of an FFN segmentation^32^ solved 57% of the splits on a random set of axons traced throughout the subvolume. This reduced the split rate from 65 to 28 splits per mm, reaching split lengths in the range required for automated connectomic analyses, while increasing merge rate from 1.7 to 3.1 per mm (in the range of merge rates for connectomic analyses as in ref. ^1^). When comparing the reduction of split errors in FFN versus RoboEM at an identical merge error rate (c2, 1.7 mergers per mm), we find that the FFN-based agglomeration from c3 to c2 only solves 8% of the split errors of c3. By contrast, RoboEM-based agglomeration solves 28% of split errors of c3 at identical merge error rate as c2 is thereby 3.5-fold more effective in split resolution than FFN (Fig. 2b). A detailed analysis of remaining axon merge errors for the two multiSEM reconstructions with RoboEM corrections furthermore showed that 86–92% of these merge errors are caused by the preceding segmentation or agglomeration, not by RoboEM itself. Further, more than or equal to 83% of major merge errors found were in close proximity to 3D data misalignment, artifacts and/or missing sections, suggesting that greater robustness to these 3D-EM data related issues could help further reduce merge errors (Supplementary Table 3 and Supplementary Note).

**Extended Data Fig. 3:**
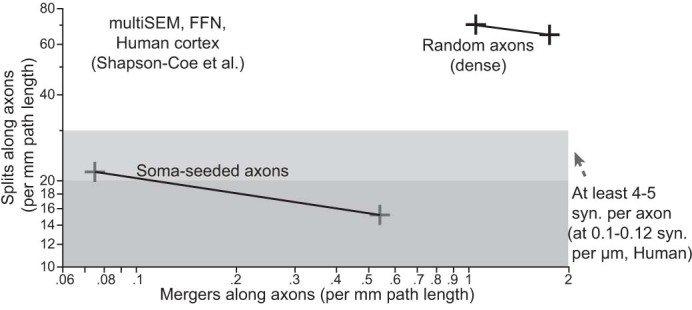
Reconstruction difficulty in proximal (soma-seeded) vs. dense (randomly-seeded) axons. Split and merge error rates for random and soma-seeded axons in the two published FFN-segmentations on multiSEM data from human cortex^18^. Note that soma-seeded axons are substantially less error-prone and therefore not representative for dense axon reconstruction performance (proximal axons are usually wider and less convoluted, thus easier to reconstruct). Source data

These results are important for two reasons. First, evaluation of automated reconstruction performance on random (‘densely seeded’) axons that are not necessarily connected to a soma within the mm^3^-scale volume provides a representative quantification of reconstruction quality for dense connectomic reconstruction. Restriction to soma-proximal axons quantifies results for soma-based proximal connectomic data, but underestimates reconstruction errors for dense axons. For example, the evaluation of FFN in human cortex, restricted to axons connected to a soma within the volume underestimates split and merge errors of random axons four- to fivefold (Extended Data Fig. 3). When using soma-based reconstructions in small brain volumes^32^, error rate quantification is highly biased to the much easier-to-reconstruct dendrites and therefore cannot be interpreted for axonal reconstructions. Second, for the connectomic analysis of axons in cortical neuropil, it is essential to obtain an interpretable number of output synapses along axons automatically. Given a certain rate of synapses along axons, this means that there is a minimum intersplit distance that needs to be achieved to obtain an interpretable number of synapses per axonal segment (Fig. 2b). The rate of synapses per axon can vary strongly between neuronal tissue types and species, such that axonal reconstructions in human cortex, for example, require at least twofold longer intersplit distances (that is, twofold lower split error rates) than in mouse cortex to achieve similar synaptic statistics per axon (Fig. 2b).

## Discussion

In summary, we find that our automated proofreading using RoboEM can fully replace human annotation for connectomic analyses of increasing difficulty and improve currently available state-of-the-art automated segmentations. This is particularly notable for segmentations obtained using FNNs^18,32^, since FFNs have a certain similarity in their design to RoboEM by recursively reconstructing individual neurites through prediction of neurite continuation from 3D-EM subvolumes. RoboEM, on the other hand, by focusing on centerline neurite tracing, may learn some aspects of axonal morphology directly and adds a notion of growth inertia to the axonal reconstruction. This enabled reconstruction problems to be solved that were not addressed by volume-based methods.

In mm^3^-scale 3D-EM datasets, the reconstruction of millimeter- to centimeter-long axons for obtaining neuron-to-neuron connectomes currently demands substantial human proofreading^18,19^. RoboEM, showing 3.5-fold better split reduction compared to state-of-the-art agglomeration by FFN^18^, allows for immediate reduction of manual-annotation needs. In addition, while we have calibrated RoboEM performance on smaller datasets (around 100–150 µm on a side), the obtained results allow us to estimate performance of RoboEM for iterative soma-seeded axonal reconstructions in larger datasets. By limiting RoboEM error correction to iterative soma-seeded axon reconstruction, and taking into account the higher accuracy on soma-proximal axons, we estimate RoboEM to extend fully automated axon reconstruction by multiple millimeters per neuron (Supplementary Note), making fully automated connectomic reconstruction at the scale of ‘peta-scale’ volumes containing thousands to ten thousand neurons in mammalian cortex and other tissue plausible.

Since RoboEM directly transforms EM image volumes into centerline tracings of neurites, it allows end-to-end learning of the key connectomic challenge: to follow axons over very long distances, also at locally thin stretches. As automated error correction framework, RoboEM allows for substantially improved reconstructions while increasing compute costs by less than 20% even compared to the recently proposed local shape descriptor framework^25^, which itself is reported to offer FFN-scale accuracy at a much reduced computational cost. With the direct end-to-end strategy, RoboEM may allow optimization for both additional accuracy and computational efficiency, which will be the next key challenge in connectomics for exabyte-scale datasets to come^58,59^.

## Methods

### Animal experiments

This section applies to the acquisition of the barrel cortex multiSEM dataset of a 28-day-old male mouse (species, *Mus musculus*, strain C57BL/6-J, one animal). The mouse cortex SBEM dataset^1^ and the human cortex multiSEM dataset^18^ analyzed in this study were taken from the respective publications.

All animal-related experimental procedures were performed according to the law of animal experimentation issued by the German Federal Government under the supervision of local ethics committees and according to the guidelines of the Max Planck Society. Experimental procedures were approved by Regierungspräsidium Darmstadt, file number V54-19c20/15-F126/1002.

The animals were bred in captivity at the animal husbandry department of the Max Planck Institute for Brain research. The room temperature was 22 °C, relative humidity 55% (±10%) and the light cycle was 12 h light/12 h dark. Autoclaved water and feed Sniff standard mouse extruded breeding or mouse/rat husbandry both ad libitum was provided. Animals were kept in breeding in type 2 long IVC greenline cages with red house and nesting material, bedding Lignocel BK8-15, under specific pathogen-free conditions.

### EM image datasets, segmentations, RoboEM training, validation and test sets

RoboEM was developed and tested on a 92.6 × 61.8 × 94.8 µm^3^ SBEM dataset from layer 4 primary somatosensory cortex of a 28-day-old mouse previously densely reconstructed^1^. The tissue was conventionally en bloc stained^60^ and imaged at 11.24 × 11.24 nm^2^ and nominal cutting thickness of 28 nm. Training and validation axons were sampled from a set of axons seeded by means of presynaptically classified segments obtained using SynEM^61^ and skeleton traced by student annotators. To also acquire a volume reconstruction of training axons, segments of the oversegmentation obtained using SegEM with parameters as set for the whole-cell segmentation of the cortex dataset^22^ were picked up and combined. The resulting volume mask was also used to iteratively optimize the interpolated skeleton tracing to yield a better centerline approximation. A set of 14 axons with 1.2-mm path lengths were used for training, and up to 0.7 million weight updates corresponding to ~260 epochs were run. The validation set consisted of 13 axons with 1.4-mm path length, where branches with less than 5 µm were excluded for better heuristic error detection. Results on the validation sets over the course of training are depicted in Extended Data Fig. 1b. A third set of ten axons with 1.7 mm path length seeded from a (2.5 µm)^3^ bounding box was used as a test set (Fig. 2b and Extended Data Fig. 1c). These are the same axons as previously used to evaluate human and semiautomated segmentation^1,42^. For automated spine head attachment on this dataset, the axon-trained RoboEM was evaluated on a random subset of 50 spine heads previously attached by human annotators, as well as on the set of spine heads previously used as a test set for semiautomated segmentation^1^.

Next, RoboEM was evaluated on a (150 µm)^3^ subvolume (dataset ID Si150L4, https://wklink.org/7122) from a 1.3 × 1.3 × 0.25 mm^3^ dataset from the barrel cortex of a 28-day-old male mouse (‘Animal experiments’ section) stained following the protocol by Hua et al.^62^ with small modifications, sectioned at 35 nm using ATUM^56^ and imaged at 4 × 4 nm^2^ using multiSEM^57^. The segmentation and agglomeration applied to the dataset at a voxel size of 8 × 8 × 35 nm^3^ (downsampled in *xy* by a factor of 2) were developed in collaboration with scalable minds GmbH, partly based on published approaches. In brief, a 3D U-Net^63^ was used to predict per cardinal axis voxel affinities^17^ from which a watershed-based oversegmentation was generated. The segments from the oversegmentation were then combined using hierarchical agglomeration^24^. In addition, neurite type predictions, blood vessel and nuclei detection were incorporated into the agglomeration to further reduce merge errors. Neurite type predictions were also used for spine head detection, which was the basis for RoboEM-based spine head attachment. For RoboEM training on axons, a training set was acquired based on a set of ten soma-seeded axons from layer 4, for which student annotators picked up segments from the oversegmentation to acquire a volume reconstruction. Here, we then used Kimimaro^64^ followed by subsampling and B-spline interpolation to extract centerline skeletons from the volume reconstruction. This yielded a training set with a total of 21-mm axon path length. As axon validation set a random ten out of 20 axons seeded from a layer 4 bounding box of size (2 µm)^3^ were traced within a (50 µm)^3^ bounding box yielding 1-mm path length. The error rate on the validation set was minimal after 3.35 million gradient updates. For the axon test set, another (1.5 µm)^3^ bounding box within layer 4 was densely annotated and a random subset of five axons were traced within the (150 µm)^3^ subvolume Si150L4 yielding 1.7 mm path length. A separate RoboEM model was trained on spine head attachment. This training and validation set was generated from a set of 20 (5 µm)^3^ bounding boxes sampled within layer 4 and annotated for spine heads. A subset of around 1,000 spine heads were volume annotated at 4 × 4 × 35 nm^3^ by student annotators from the spine head through the spine neck up to the dendritic trunk. Here, we again used Kimimaro^64^ to extract centerline skeletons from the volumetric masks for training yielding 2 mm of spine neck tracings. Additionally, a random subset of 76 spine heads with 0.2-mm path length from the 20 bounding boxes was skeleton traced to serve as validation set on which the error was minimal after 0.9 million gradient updates. The evaluation was done on another randomly selected (5 µm)^3^ bounding box (Si11L3 https://wklink.org/2458) containing 91 densely annotated spine head to dendritic trunk skeleton tracings.

For the evaluation of RoboEM-based error correction of existing state-of-the-art segmentation, we applied RoboEM to a published mm^3^-scale multiSEM dataset^18^ from human cortex with voxel size 4 × 4 × 33 nm^3^ segmented and agglomerated using FFNs^32^. Specifically, we focused on a (150 µm)^3^ bounding box containing 6.5 mm of axon path length of the provided ground truth skeleton tracings. The published ground truth skeleton tracings in this box were then used to evaluate FFN on soma-seeded axons^18^ (Extended Data Fig. 3). To finetune RoboEM on this dataset, we generated ground truth skeleton tracings of dense seeded axons by sampling a bounding box of size (2.5 µm)^3^ within a centered (15 µm)^3^ bounding box, annotating all processes in this bounding box and then sampling a random subset of five axons, which were traced throughout the (150 µm)^3^ bounding box yielding 1.25 mm path length. Here, skeleton annotations were already done with high precision along the centerline such that no postprocessing was necessary. The volumetric neurite mask needed for training was generated by means of segment pick-up from the c3 FFN segmentation^18^. The best RoboEM model checkpoint, based on the validation set from the axon training in the mouse cortex ATUM-multiSEM dataset (subvolume Si150L4), was then used as initialization and RoboEM was trained for another 1.95 million gradient updates until converging in terms of reset-based error rates on the training axons. For the evaluation of FFN and RoboEM another bounding box of size (1.5 µm)^3^ was annotated for a random subset of five axons traced throughout the (150 µm)^3^ bounding box of the human cortex multiSEM dataset^18^, yielding 1.4 mm path length.

### Neurite flight reconstruction

We phrase the problem of neurite reconstruction from a 3D-EM volume as a centerline reconstruction task, in which a neurite is represented by a sequence of visited points. A CNN^65^ is trained on the task of predicting the local neurite continuation from a neurite-centered and -aligned 3D-EM subvolume (Fig. 1d,e and Extended Data Fig. 1a) similar to human annotation flight mode^42^. Integration of the predicted neurite continuation yields a new position and orientation, which is used to generate the subsequent CNN input. Iterative application of this procedure turns a starting location and orientation into a neurite skeleton reconstruction using only 3D-EM data and without intermediary steps, such as volume segmentation.

The input to the CNN consists of a 96 × 96 × 16 voxel neurite-centered and -aligned 3D-EM subvolume that covers, for example for axons, a field of view of ~1 × 1 × 0.7 µm^3^ (Fig. 1e and Extended Data Fig. 1a). The third (*z*) dimension corresponds to the current flight direction, while the current position is made the center of the fourth *z* plane. The field of view is thus asymmetric along the *z* direction with more contextual information available in forward than in reverse flight direction. Empirically, this allows for better steering toward the ‘exit’ within axonal varicosities. The EM data is projected onto neurite-aligned planes by means of trilinear interpolation.

For the CNN architecture, we use seven 3D strided convolutional layers, followed by a dropout layer (dropout rate 0.5), three fully connected layers and a last linear layer that estimates the two steering commands and the distance to membrane along the flight direction (Extended Data Fig. 1a). The two steering commands are the Bishop curvatures^54^ described in more detail below. A two-dimensional CNN architecture previously proposed for image-based road following^53^ served as a starting point for the architecture in this work. As nonlinearity we tested rectified linear units^66^ (ReLU) and exponential linear units^67^ (ELU), and found ELU to work better (Extended Data Fig. 1b).

For the mathematical formulation of the network’s input and output, the Bishop frame, a local orthonormal coordinate system spanned by a tangential vector **t** and two normal vectors **n**_1_ and **n**_2_ and obeying a rotation minimizing property, and the associated Bishop curvatures *k*_1_ and *k*_2_, were found to be particularly suitable^54^ (Fig. 1d and Extended Data Fig. 1a). Here, we define the neurite-aligned projection planes for the network’s input by means of the normal Bishop vectors, while the network’s flight direction corresponds to the tangential vector. The evolution of the Bishop frame unit vectors is coupled via the Bishop curvatures *k*_1_ and *k*_2_, one for each normal vector direction and corresponding to signed curvatures for steering toward left to right and up and down, respectively. Note that the signed curvature has previously been used as steering command for left to right steering in image-based road following limited to planar curves^53^. The task of predicting the neurite continuation in the form of Bishop curvatures can be interpreted as fitting a parabola to the neurite’s centerline, where the curvature vector $\,{\bf{k}}={k}_{1}{\bf{n}}_{1}+{k}_{2}{\bf{n}}_{2}$, determines direction and magnitude of bending of this parabola (Fig. 1d and Extended Data Fig. 1a).

### Training and inference

For training the above-described CNN architecture on the neurite-following task, we implemented the CNN in TensorFlow^68^ and trained with a mini-batch size of 128 using RMSProp^69^ with momentum^70,71^, for the SBEM dataset^1^ and Adam^72^, for the multiSEM datasets, on minimizing the mean-squared error plus a L2 regularization loss term on the weights. Weights for layers with ReLU or ELU activations were initialized following He et al.^73^, and the final layer was initialized following Glorot et al.^74^.

Similar to a finding by Pomerleau^52^ for image-based road following, we also found that training on the neurite centerline alone does not yield good generalization performance during inference, as current position and orientation depend on past network decisions and errors can accumulate. To achieve stable path following, we trained the CNN on off-centerline positions and off directions with correspondingly adjusted steering leading back to the neurite centerline, where we refer to the mapping from a particular off position, off-direction state to the adapted steering as the flight policy.

We derived a greedy flight policy based only on local information available to the CNN within the finite field of view (details below). We induced the notion of obstacle (that is, the membrane) avoidance through the usage of a dynamic convergence distance set to the distance to the plasma membrane along the flight direction, which empirically allowed for better performance than a constant value. Additionally, we used the distance to plasma membrane as an auxiliary loss term during training.

To reconstruct neurites during inference, the steering predictions of the CNN for a position and orientation are integrated to a new position and orientation used to generate the subsequent input (Extended Data Fig. 1a, normal inference). For the random rotation inference mode (Extended Data Fig. 1a) we additionally perform a random rotation around the tangential after the integration step, which decorrelates consecutive inputs and comes at negligible computational cost. In both inference modes, given a start position and orientation, the recurrent application of the CNN yields a trace of visited points corresponding to the network’s prediction of the neurite centerline.

Whereas for an error correction framework, such as FocusEM^1^, both start position and orientation can be provided, for some use cases the orientation might not be available. Specifically, for the task of attaching spine heads to their corresponding dendrites, it can be less obvious how to compute the start orientation. To apply RoboEM on spine neck tracing tasks given only the start positions, we run stochastic forward passes through the dropout layer, known as Monte Carlo dropout^75,76^, yielding a sampled distribution of predictions, from which we estimate prediction uncertainty. The uncertainty can then be used to select a start orientation from a list of candidate orientations or rank tracings thereby allowing for a trading off split against merge errors. As there is only a single dropout layer after the convolutional layers (Extended Data Fig. 1a), the computation for different dropout masks is shared up to this point and the overhead of Monte Carlo dropout is negligible (<2% in terms of floating point operations for 128 Monte Carlo samples).

### B-spline interpolation and RoboEM step size

To get a continuous representation of neurite branches from sparsely placed nodes from human skeleton reconstructions, we use degree 4 B-spline interpolation^77^ yielding a curve ${\bf{\gamma }}\in {C}^{3}$. We reparametrize the curve to get a curvature adaptive step size $\Vert \dot{\bf{\gamma }}\,\Delta t\Vert$ of

$$\Vert \dot{\bf{\gamma }}\Vert \Delta t=\,f \frac{d}{1+\frac{p}{2}\kappa },$$

where $\kappa =\Vert {\bf{k}}\Vert$ is the curvature, *p* = 1 µm the physical size of the projection plane and *f* is a step size factor set to *f* = 1 for training that can be adjusted to higher values for inference. While for most evaluations in this work, we also used *f* = 1 during inference, step sizes can be increased up to *f* = 5 yielding correspondingly increased throughput without sacrificing accuracy (Extended Data Fig. 1c). A default step size *d* matching the smallest dimension of a voxel, such as *d* = 11.24 nm in case of the L4 SBEM dataset^1^ at *f* = 1 ensures that no voxels up to a radius of half the projection plane size in the projection plane at the current position of the CNN are skipped. To keep the notation uncluttered, subsequent formulas are expressed in terms of the arc length parametrized curve ${\bf{\gamma }}(s)$ indicated by the parameter *s*.

### Bishop frame

The Bishop frame^54^ consists of three orthonormal vectors, namely the tangential vector **t**, in this work also termed flight direction, and two normal vectors **n**_1_, **n**_2_. The evolution equations of the Bishop frame and the curve ${\bf{\gamma }}$ read as follows:

$$\frac{{\rm{d}}}{{\rm{d}}s}{\bf{\gamma }}={\bf{t}}$$

$$\frac{{\rm{d}}}{{\rm{d}}s}{\bf{t}}={k}_{1}{\bf{n}}_{1}+{k}_{2}{\bf{n}}_{2}\equiv {\bf{k}}$$

$$\frac{{\rm{d}}}{{\rm{d}}s}{\bf{n}}_{1}=-{k}_{1}\bf{t}$$

$$\frac{{\rm{d}}}{{\rm{d}}s}{\bf{n}}_{2}=-{k}_{2}{\bf{t}}.$$

Here, *k*_1_ and *k*_2_ are the Bishop curvatures associated to the normal vector **n**_1_ and **n**_2_, respectively, and **k** is the curvature vector (Fig. 1d and Extended Data Fig. 1a). Flips and rotations around the tangential vector applied to both normal vectors and Bishop curvatures are invariance transformations that leave **k** and thereby the evolution of ${\bf{\gamma }}$ and **t** unchanged and are used for data augmentation during training.

The rotation minimizing Bishop frame has weaker requirements for a parameterized curve ${\bf{\gamma }}$ than the Frenet–Serret frame often used as local coordinate system in differential geometry of parameterized curves. Specifically, the Bishop frame of a parametrized curve in 3D Euclidean space does not require *κ* ≠ 0, and only requires ${\bf{\gamma }}\in {C}^{2}$ instead of ${\bf{\gamma }}\in {C}^{3}$: for details, see ref. ^54^. Initial conditions in form of start position and Bishop frame orientation together with the Bishop curvatures uniquely define the centerline curve ${\bf{\gamma }}$. The integration of above Bishop equations during inference was performed with either of two methods: (1) forward Euler method followed by Gram–Schmidt orthonormalization to maintain an orthonormal basis for the Bishop frame, or (2) analytically derived evolution equations for a Bishop frame along a parabola. Empirically, we found the latter to work better especially for larger step size factors *f* (Extended Data Fig. 1c).

### Flight policy

To derive a flight policy for stable path following during inference, the Taylor series expansion up to second order ${\bf{\gamma }}_{T}$ for the known neurite centerline curve ${\bf{\gamma }}(s)$ with corresponding Bishop frame **t**, **n**_1_, **n**_2_ and curvature vector **k**, and the Taylor series expansion up to second order ${\underline{\bf{\gamma }}}_{T}$ of the unknown off-centerline, off-direction curve $\underline{\bf{\gamma}}(s)$, with **t**, **n**_1_, **n**_2_, **k**:

$${\bf{\gamma }}_{T}(s)={\bf{\gamma }}+{{s}}\;{\bf{t}}+\frac{{{s}}^{\mathrm{2}}}{\mathrm{2}}{\bf{k}}$$

$${\underline{\bf{\gamma }}}_{T}(s)={\underline{\bf{\gamma }}}+s\;{\underline{\bf{t}}}+\frac{{s}^{2}}{2}\underline{\bf{k}}$$

were used.

Knowing the current off position $\underline{\bf{\gamma}}$ and off-direction **t**, as well as the current closest position on the neurite centerline ${\bf{\gamma}}$ with correct neurite-aligned orientation **t** and local shape of the curve in terms of **k**, that is correct steering for the on centerline case, we derived suitable corrected steering **k** converging back to the centerline within some distance *s*_c_, that is, which minimizes the future distance $\Vert {\bf{\Gamma}}_{T}\Vert$ defined by:

$$\underbrace{{\mathbf{\gamma }}_{T}-{\underline{\mathbf{\gamma }}}_{T}}_{{\mathbf{\Gamma }}_{T}}=\underbrace{\mathbf{\gamma }-\underline{\mathbf{\gamma }}}_{\mathbf{\Gamma }}+s\underbrace{(\mathbf{t}-\underline{\mathbf{t}})}_{\mathbf{{{T}}}}+\frac{{s}^{2}}{2}\underbrace{(\mathbf{k}-\underline{\mathbf{k}})}_{{{\mathbf{K}}}}.$$

We therefore require:

$${\nabla }_{\underline{\bf{k}}}{\Vert {\bf{\Gamma }}_{T}({s}_{\mathrm{c}})\Vert }^{2}=0,$$

where ${\nabla }_{\underline{\bf{k}}}={\underline{\bf{n}}}_{1}{\partial }_{{\underline{k}}_{1}}+{\underline{\bf{n}}}_{2}{\partial }_{{\underline{k}}_{2}}$. Defining the projection operator ${{\mathscr{P}}}_{{\underline{\bf{n}}}_{1},{\underline{\bf{n}}}_{2}}=$ ${\underline{\bf{n}}}_{1}\otimes {\underline{\bf{n}}}_{1}+{\underline{\bf{n}}}_{2}\otimes {\underline{\bf{n}}}_{2}$, where ⊗ denotes an outer product, the solution for $\underline{\bf{k}}$ from the above equation reads as follows:

$$\underline{\bf{k}}={{\mathscr{P}}}_{{\underline{\bf{n}}}_{1},{\underline{\bf{n}}}_{2}}\left(\frac{2}{{s}_{c}^{2}}[{\bf{\Gamma }}+{s}_{\mathrm{c}}{\bf{t}}]+{\bf{k}}\right).$$

Iterative application with updated values for ${\bf{\gamma }}_{T}$ at the closest point ${\bf{\gamma }}$ from the current position ${\underline{\bf{\gamma}}}$ yields a stable path following flight policy with a free parameter *s*_c_ controlling the convergence speed. Trajectories for different values of *s*_c_ are plotted in Extended Data Fig. 1b. For this work, during training, we set *s*_c_ to the current distance to membrane along the flight direction and thereby train RoboEM on a membrane avoidance strategy.

### Direction prediction with Monte Carlo dropout

For the direction prediction, as for example needed for spine head attachment where only an initial position is given, we sample *I* = 256 roughly equidistant orientations ${\bf{t}}_{i}$ and run Monte Carlo dropout^75^ with *M* = 128 samples. From the sampled Bishop curvature predictions ${\bf{k}}_{im}$, we compute the mean curvatures 〈*κ*〉_*i*_ and the covariances of the Bishop curvatures cov_*i*_ per orientation *i* over the Monte Carlo samples. The uncertainty estimate *u*_*i*_ is then taken as the square root of the largest eigenvalue of the covariance matrix divided by the mean curvature:

$${u}_{i}=\sqrt{\max ({\mathrm{eigvals}}({\mathrm{cov}}_{i}))}/{\left\langle \kappa \right\rangle }_{i}.$$

Using cosine similarity for neighboring angles within 30°, we construct a weighted average of uncertainties ${\widehat{u}}_{i}$ as

$${\widehat{u}}_{i}=\frac{{u}_{i}}{2}+\frac{1}{2}\mathop{\sum}\limits_{j}{\widehat{w}}_{{ij}}{u}_{i}$$

with weights $${\widehat{w}}_{{ij}}$$

$${\widehat{w}}_{ij}={w}_{ij}/\mathop{\sum }\limits_{j}{w}_{ij}$$

$${w}_{ij}=\left\{\begin{array}{ll}{\langle {\bf{t}}_{i}|{\bf{t}}_{j}\rangle }^{16} & {\rm{if}}\,\arccos (\langle {\bf{t}}_{i}|{\bf{t}}_{j}\rangle ) < 30^\circ \\ 0 & {\rm{otherwise}}\end{array}\right.$$

to give more stable predictions. The orientation with minimal averaged uncertainty ${\widehat{u}}_{i}$ was used as first orientation candidate. For spine head attachment in the mouse cortex ATUM-multiSEM dataset (subvolume Si11L3), we used a second candidate that was >110° from the first and has again minimal averaged uncertainty among the remaining orientations.

### Model selection from validation set

To choose a model from a pool of trained models with different learning rates, fields of view and so on, we evaluated RoboEM on a validation set consisting of human skeleton annotations without any segmentation. Specifically, RoboEM was evaluated on linear neurite branches by running recurrent inference starting from both sides. In case RoboEM reaches a first set of thresholds (Supplementary Table 1) concerning the distance or the angle with respect to the ground truth tracing, we consider the tracing to be ‘experimental’, that is the progress along the ground truth tracing is temporarily not considered until the first set of thresholds is not exceeded anymore. If a second set of thresholds (Supplementary Table 1) is exceeded, the tracing is classified as erroneous and a reset to the closest point on the ground truth before the tracing turned experimental is performed. Tracing is stopped, when the closest point on the ground truth is at the other end of the axonal branch and the tracing is at that time classified as correct.

To relate resets caused by steering errors to merge and split error rates, we consider each reset both a merge error into a wrong process and a split error due to not continuing the neurite of interest. Hence, we count each reset as two errors and report this as a reset-based error rate. While this cannot be used to directly compare with segmentation error rates, it serves as a metric for model selection.

For model selection on the SBEM L4 dataset^1^, we find that averaged over all models and training iterations the random inference mode outperforms its normal mode counterpart by 33% (range 12–52%). The best performing model on the validation set uses only EM data as input, has an ELU activation function, was trained for 700,000 training iterations and yielded 17 errors per mm (Extended Data Fig. 1b).

### Split and merge error evaluation

For the evaluation of split and merge errors of agglomerations before and after RoboEM-based error correction (Fig. 2 and Extended Data Fig. 3), we detected merge errors that extended further than 2.2 µm from the ground truth and manually verified that this heuristic accurately detects merge errors. For agglomerations before RoboEM corrections, each merge error was counted as 0.5 and divided by the ground truth path length to yield the merge error rate. This is because each merge error usually connects two neurites and counting them as one error per neurite instead of 0.5 would overestimate the total amount of merge errors. For sparse evaluations of RoboEM-based error corrections limited to agglomerates that overlap with the ground truth, as done in both multiSEM datasets evaluated in this work, additional mergers introduced by RoboEM were counted as one instead of 0.5, which accounts for mergers from agglomerates not overlapping with ground truth and therefore not observable in a sparse evaluation. For split errors, we restricted the set of agglomerates to be evaluated to those agglomerates that overlap more than 2.5 µm with the ground truth. We introduced this overlap threshold to avoid domination of split errors by many small agglomerates or unagglomerated segments along thin stretches of axons. Note that despite this overlap length threshold, around 90% of the ground truth was still covered.

For the multiSEM datasets, RoboEM-based correction was restricted to ending resolution, for which endings were extracted from skeleton representations of those agglomerates that overlapped with the ground truth annotations. In addition to the RoboEM validation strategy, we also made use of subcellular type predictions to decide whether a RoboEM tracing should connect two agglomerates. Note that type predictions were also used by FFN to avoid merge errors across subcellular types^18^. After agglomerates were reconnected using RoboEM tracings, the resulting agglomeration state was evaluated as before.

To test whether RoboEM-based correction also allows for reduced split error rates when compared at the same merge error rate as the segmentation and/or agglomeration for the two multiSEM datasets, the following strategy was used to only apply subsets of RoboEM tracings thereby limiting merge error rates: prediction uncertainty using Monte Carlo dropout was quantified for every RoboEM step, then the maximum uncertainty over steps was taken and forward and validation tracings were combined with a minimum of the two uncertainty scores. The rationale was to first score every direction of the validated tracings according to the position with maximum uncertainty and then, since the forward and backward tracings yielded the same flight path, the minimum of those two uncertainty scores was taken to quantify the uncertainty of the tracing as a whole. Application of validated RoboEM tracings up to percentiles of 0.2, 0.4, 0.6 and 0.8 of tracing uncertainties then yielded partial RoboEM corrections. These were evaluated for split and merge error rates and those with minimal split error rates at same merge error rate as segmentations/agglomerations were added as intermediate points within the split merge error plane of Fig. 2b. Specifically, for the mouse cortex multiSEM dataset, application of the top 80% of validated RoboEM tracings on the agglomeration at 85% agglomeration threshold yielded the same merge error rate as the agglomeration at 90% agglomeration threshold. Similarly for the human cortex multiSEM dataset, the application of the top 40% of validated RoboEM tracings on the FFN c3 agglomeration yielded the same merge error rate as the FFN c2 agglomeration^18^.

### Reporting summary

Further information on research design is available in the Nature Portfolio Reporting Summary linked to this article.

## Online content

Any methods, additional references, Nature Portfolio reporting summaries, source data, extended data, supplementary information, acknowledgements, peer review information; details of author contributions and competing interests; and statements of data and code availability are available at 10.1038/s41592-024-02226-5.

### Supplementary information

Supplementary Information Supplementary Note, Fig. 1 and Tables 1–4.

Reporting Summary

Supplementary Video 1 Flight tracing of a branch of a test set axon from the SBEM dataset (Motta et al.^1^; Fig. 2b, length of axon: 60 µm). This axon was not used during training of RoboEM. Visualized tracing speed was 3.4 mm per hour (actual tracing speed when parallelized over multiple tasks; Methods and Supplementary Note, roughly 160 mm per hour). Left, the view along cutting direction of 3D-EM data; right, a two-dimensional cutout of the field of view of RoboEM at the current tracing position, oriented perpendicular to the flight path (image plane in Extended Data Fig. 1a with red outline).

Supplementary Code 1 Code for training and inference of RoboEM and for the analyses performed in this study.

### Source data

Source Data Fig. 1 An .xlsx file for split and/or merge error resolution (Fig. 1g) and compute costs (Fig. 1i).

Source Data Fig. 2 An .xlsx file for split and/or merge error rates (Fig. 2b).

Source Data Extended Data Fig. 1 An .xlsx file for reset-based error rates on the validation set over training iterations (Extended Data Fig. 1b) and for reset-based error rates on the mouse cortex SBEM axon test set for Euler versus Parabola integration and different step size factors (Extended Data Fig. 1c).

Source Data Extended Data Fig. 3 An .xlsx file for split and merge error rates of FFNs on soma-seeded and random (dense) axons in the human cortex multiSEM dataset.

## Data Availability

All data necessary to reproduce reported results for the mouse cortex SBEM dataset^1^ are available: https://wklink.org/9276 (raw data), https://L4dense2019.brain.mpg.de (code and data of the original publication); trained RoboEM weights, and evaluation data are available in the supplement subject to provisions as stated in the code availability section (below). Raw data for the mouse cortex multiSEM dataset are available: Si11L3 https://wklink.org/2458 (spine head attachment test set), Si150L4 https://wklink.org/7122 (axon test set); RoboEM training data for the mouse cortex multiSEM dataset are available: https://wklink.org/8172. Raw data and training data for the human cortex multiSEM dataset^18^ were obtained from and are available as detailed in ref. ^18^ at https://h01-release.storage.googleapis.com/landing.html. Trained RoboEM weights for mouse and human multiSEM data are available on reasonable request to allow usage monitoring according to licensing criteria (all source code and binary files are publicly available under the limited right to use for the exclusive purpose of undertaking academic or not-for-profit research, as further detailed in the code availability section below). In addition, data needed to run the example code is part of the zipped code package provided in the supplementary material subject to provisions as stated in the code availability section (below). Source data are provided with this paper.

## Code availability

All source code and binary files are publicly available in the supplement under the following provisions: any copyright or patent right is owned by and proprietary material of the Max Planck-Gesellschaft zur Förderung der Wissenschaften e.V. (MPG). MPG makes no representations or warranties of any kind concerning the source code and binary files, neither express nor implied and the absence of any legal or actual defects, whether discoverable or not. The source code and binary files available in the supplement are subject to a non-exclusive, revocable, non-transferable, and limited right to use for the exclusive purpose of undertaking academic or not-for-profit research. Use of the Code or binary files or any part thereof for commercial or clinical purposes and alterations are strictly prohibited in the absence of a Commercial License Agreement from MPG. Download and/or use of the source code and binary files under the aforementioned research license comes with the acceptance of the license agreement provided with the download.
