# Supplementary material for: RoboEM: automated 3D flight tracing for synaptic-resolution connectomics
Source: Nat Methods. 2024 Mar 21;21(5):908–13. doi: 10.1038/s41592-024-02226-5 (PMC11093750; doi:10.1038/s41592-024-02226-5)
Supplement: Supplementary file 1 — Supplementary Note, Fig. 1 and Tables 1–4. [file 41592_2024_2226_MOESM1_ESM.pdf]

nature methods

Article

https://doi.org/10.1038/s41592-024-02226-5

RoboEM: automated 3D flight tracing for synaptic-resolution connectomics

In the format provided by the authors and unedited

CONTENTS

1 Supplementary Note 2

1.1 RoboEM as error correction framework 2

1.2 Maximum error rates for the study of axonal synaptic properties 4

1.3 Assessment of remaining merge errors 5

1.4 Computational cost 6

1.5 Estimate of RoboEM performance for soma-based iterative axon reconstruction in mm^3-scale datasets 7

2 Supplementary Figure 9

3 Supplementary Tables 10

4 References 13

1 SUPPLEMENTARY NOTE

1.1 RoboEM as error correction framework

To show that RoboEM enables fully-automated connectomic reconstructions of relevant volumes when used as an error correction framework, we reran the dense reconstruction of (Motta et al., 2019) using RoboEM as a direct replacement for human annotations within the FocusEM framework. To this end, we trained RoboEM on axons (see Methods) and applied it to axon and spine neck reconstruction.

Specifically, we ran RoboEM on all detected spine heads that previously required human annotations (38% of all detections consuming around 900 working hours of annotators) and found that this yields an improvement of recall from automated methods as used in (Motta et al., 2019) from 58% to 70%, while precision decreased from 93% to 85%, which compares to 96%/91% when using human annotations. In detail, we used the start positions from the spine head detection also provided to human annotators and first ran the direction prediction via Monte Carlo Dropout (see Methods). We then applied the recurrent inference mode of RoboEM for the first candidate direction with lowest uncertainty and traced until a dendritic trunk defined by a dendrite mask was reached or a path length threshold was exceeded. Notably, RoboEM has not been retrained on spine necks for this dataset, such that further improvements can likely be achieved by assembling a dedicated training set for spine necks as has been done for the mouse cortex multiSEM dataset.

For the correction of split and merge errors in the axon reconstruction, which previously consumed 3000 working hours of annotators, we supplied start positions and directions from the FocusEM framework to RoboEM. For split error resolution, RoboEM was iteratively run on stretches of 1.5 µm and each stretch was validated by running RoboEM backwards. Only if the validation was successful, the tracing continued with the next stretch until a known axon agglomerate or the end of the dataset was reached. The resulting skeleton tracings could then be used analogous to human annotations. For merge error resolution, the stop criterion was based on a bounding box around the merge error – again analogous to human – and tracings were accepted if validation in backward direction yielded the same skeleton reconstruction with some error tolerance. Split error resolution was run once

(~128,000 ending queries) and flight paths, for which a new agglomerate was found and the full path length was validated, were incorporated (~60% of all ending queries). Next, three rounds of merge error resolution were run until less than 50% of chiasmata were solved by RoboEM annotations (first round: ~8,100 chiasmata, ~4,400 solved; second round: 4,100 chiasmata, 3,300 solved; third round: ~1,000 chiasmata, ~300 solved). Finally, partially validated flight paths from the first split error resolution were added to the agglomerates and segments that only overlap with flight paths from a single agglomerate were added to the respective agglomerates. The final axon reconstruction for this fully automated approach yielded 12 split errors/mm and 8 merge errors/mm on the test set axons comparing to 5 split errors/mm and 6 merge errors/mm for the semi-automated reconstruction, cf. Fig. 2b.

Using the fully automated reconstruction acquired by previous automated methods and RoboEM, we reran parts of the biological analysis as done by (Motta et al., 2019). This allowed us to compare: (CI) the connectome from fully automated reconstruction prior to human annotations, (CII) the connectome from the semi-automated reconstruction including 4,000 work hours of human annotation, (CIII) the connectome obtained from combining the fully automated reconstruction CI with RoboEM split and merge error corrections yielding an automatically proofread connectome. Resulting figures for the three reconstruction states, such as number of axons, synapses etc. are summarized in Suppl. Table 2. Results of the paired same-axons same-dendrite analysis are shown in Extended Data Fig. 2a. While there are many more synapse pairs recovered for the reconstruction states obtained by human and RoboEM-based correction (Number of same axon same dendrite pairs (I) n = 993, (II) n = 5290, (III) n = 3982), the resulting fractions of paired connections consistent with long term potentiation (LTP) is similar across states ((I) 11-20%, (II) 16-20%, (III) 13-19%). In contrast, the spine densities prior to corrections are 1.4-fold lower for apical dendrites than after human proofreading, while RoboEM-based correction yields 1.08-fold lower spines per µm, cf. Extended Data Fig. 2b. Further, when distinguishing excitatory and inhibitory axons with ≥10 synapses based on their fraction of primary spine innervation, in the automated agglomeration there are 62% fewer axons reaching the synapse number threshold compared to the final reconstruction using human annotations. Additionally, the resulting proportion of excitatory versus inhibitory axons is skewed yielding only 75% excitatory axons (estimate from (Motta et al., 2019): 87%;

cf. Extended Data Fig. 2c). RoboEM-based correction of split and merge errors not only recovers 97% of axons reaching the synapse number threshold, but also yields a better estimate of the proportion of excitatory to inhibitory axons of 84%, cf. Extended Data Fig. 2c. Finally, target specificities of axons were evaluated across reconstruction states against a binomial null model. While the overall fractions of specific axons are similar across reconstruction states (fraction of exc. axons specific for false detection rate thresholds 5-30%: (I) 9-35%, (II) 9-33%, (III) 6%-30%; fraction of inh. axons specific: (I) 29-55%, (II) 38-62%, (III) 45-69%, cf. Extended Data Fig. 2d), the automated reconstruction prior to human or RoboEM-based correction fails to recover apical and smooth dendrite specificities of inhibitory axons, cf. Extended Data Fig. 2d.

1.2 Maximum error rates for the study of axonal synaptic properties

Studying axonal synaptic properties requires a minimum number of synapses per reconstructed axon fragment (depending on the type of analysis), which in turn translates into a maximum split rate / minimum split-free path length that can be tolerated, depending on the species-specific synapse density along axons. For intermediate-scale connectomic analyses in mouse cortex, as performed by (Motta et al., 2019), a threshold of ≥10 synapses per axon fragment, corresponding to ≥50 µm inter split distance and ≤20 splits per mm (at synapse densities of ~0.2 per µm axon path length), allowed for a clear separation of excitatory and inhibitory axons based on their primary spine innervation profile and was used for subsequent analyses of other axonal synaptic properties. The reconstruction state from (Motta et al., 2019) prior to human or RoboEM-based error correction (with an error rate of >30 splits per mm, (Fig. 2b)), using a synapse count threshold of at least 10 per axon leads to an underestimation of excitatory versus inhibitory axons and similarly does not allow to recover inhibitory axon specificities for apical and smooth dendrites (Extended Data Fig. 2c,d). This is in contrast to the RoboEM- and human-error corrected reconstructions with <30 splits per mm (Fig. 2b, Extended Data Fig. 2c,d).

To obtain a more quantitative estimate of required error lengths also for human data, we used a more detailed analysis of the discriminability of excitatory and inhibitory axon fragments in mouse, macaque and human cortex reported in (Loomba et al., 2022). There, using Bayesian modeling, the number of spine and shaft synapses along axonal fragments was related to the probability for an axon fragment to be excitatory

or inhibitory. Here, we amended the validation experiments shown in Fig. S2 of (Loomba et al., 2022), where posterior probabilities for axon fragments with 10 synapses of known type to be excitatory or inhibitory were computed based on optimized Bayesian model parameters, the number of shaft and spine synapses, as well as the postsynaptic type for 1/10 synapses. Specifically, using both data and the model from (Loomba et al., 2022), we recomputed the type predictions for fragments with 2-10 synapses and computed the expected misclassification rate for mouse versus macaque/human, finding that a distinction between excitatory and inhibitory axons is already possible based on axon fragments with 2-3 synapses for mouse cortical axons, while macaque/human cortical axons require at least 4 synapses for misclassification rates of ≤25%, and at least 8 synapses for misclassification rates ≤10% (Suppl. Fig. 1).

Based on above considerations, in human cortex ≥75% accurate identification of excitatory and inhibitory axons requires ≥4 synapses (~0.1-0.12 synapses per µm axon path length) corresponding to ≤30 splits per mm and ≥33 µm inter split distance. The same inter split distance in mouse cortex allows already for more detailed analyses, as, e.g., the study of inhibitory axon target specificities.

1.3 Assessment of remaining merge errors

The kinds of remaining merge errors after RoboEM-based correction differ for the two multiSEM segmentation/agglomeration pipelines, cf. Suppl. Table 3. To characterize merge errors, we first distinguished between minor and major merge errors. Minor merge errors have ≤1µm overlap with the ground truth axon skeleton annotation and therefore are unlikely to wrongly assign presynaptic axonal boutons along the ground truth axon to neighboring processes. Indeed, we did not find a single synaptic bouton along the ground truth axon for agglomerates with minor merge errors. In the multiSEM human cortex (Shapson-Coe et al., 2021) FFN c2 reconstruction after RoboEM-based correction only 14% of merge errors are minor merge errors. In contrast, in the multiSEM mouse cortex dataset (Si150L4), minor merge errors make up 77% (10/13) and are themselves dominated by axon-glia merge errors (7/10) at locations where the axons are very thin (≤100 nm) and in individual sections not clearly identifiable as a separate process. In all cases of minor merge errors, the errors were not caused by RoboEM and instead pre-existed either already in the oversegmentation (90% for

multiSEM mouse cortex dataset), or were introduced in the agglomeration. Therefore, the resolution of minor merge errors is in most cases ideally solved on the level of the oversegmentation, however, is only critical if those merge errors act as nucleus of crystallization for major merge errors upon further reduction of split errors (in case of RoboEM-based split error correction, we only found one such case).

Among major merge errors with >1μm of overlap with the ground truth skeleton annotation, we further checked, if 3D-EM data related issues, such as misalignment, artifacts, missing sections, or any combination thereof are in close spatial proximity to major merge errors. Indeed, most of major merge errors (83-100%) could be associated to those putative causes, cf. Suppl. Table 3. Since in all observed cases, 3D-EM data was unambiguous enough for human experts, an automated resolution of those errors by improvements in alignment quality and robustness of reconstruction methods against artifacts seems plausible.

The only example of a major merge error without an identified image data related cause involves two thin parallel axons. Specifically, the ending of a ≤100nm thin axon is merged into a similarly thin neighboring axon by a RoboEM flight path at a location, where only membranes and no lumen of the two axons are visible and therefore they are not clearly separable in this image plane even by human experts who relied on direction and axon diameter cues to find the most likely continuation.

1.4 Computational cost

To estimate computational costs, we benchmarked RoboEM on the ~128,000 ending tasks from the first set of ending detections in (Motta et al., 2019). The step size factor was set to $f = 5$ and analytically derived equations for the Bishop frame along a parabola were used to integrate steering predictions. This is in contrast to other evaluations presented in this work, which used $f = 1$ and integration using the forward Euler method (for the validity of increasing step size and hence throughput, cf. Extended Data Fig. 1c). The total runtime on a single node using 32 cores (Intel(R) Xeon(R) Gold 6130 CPU, 2 sockets), a single Tesla V100 GPU (PCIE-16GB) and less than 128GB RAM was 13.6 hours for the reconstruction of around 2.1 meters of axons (including backward validation tracings, a total of 64 million CNN inferences), and hence a reconstruction speed of around 160 mm/h (~1300 steps/s, average step size of 33 nm). From this and previous RoboEM runs with $f = 1$ and integration using the

forward Euler method, we extrapolated to yield a total runtime of 27.9 hours for axon and spine neck tracing (4.3 meters including path length for validations), and 7.6 hours for initial direction prediction (on ~138,000 spine heads) on a single node for the automated error correction of the reconstruction state from (Motta et al., 2019) before human interventions yielding a total of 35 single GPU node hours. For flood-filling networks the 6.964 GPU node hours were multiplied by the 1000 (NVIDIA P100) GPUs (Januszewski et al., 2018). For Local Shape Descriptors (LSD), the AcRLSD architecture was reported to consume a total of 10.5 hours on 24 V100 GPU nodes, watershed and agglomeration was reported to consume 7.7 CPU node hours with 100 cores (Sheridan et al., 2022). For (Motta et al., 2019), the dense reconstruction including segmentation, agglomeration, type and synapse prediction and processing of human skeleton reconstructions took 101 hours on 24 nodes with 16 CPU cores each. At the time of writing, Amazon EC2 (x2gd.8xlarge instance) costs per CPU core are at 0.0835 USD/h and a single T4 GPU node with 32 cores and 128GB RAM (g4dn.8xlarge) costs 2.176 USD/h. Since T4 GPUs only have 8 TFLOP (single precision) in comparison to 14 TFLOP on V100 GPUs and 11 TFLOP on P100 GPUs, we adapted the costs accordingly. For all methods the costs were multiplied by the ratio of the respective dataset sizes and normalized to the 2.7 meters of neurites (Motta et al., 2019), cf. Suppl. Table 4, resulting in the compute cost estimates in Fig. 1h,i (using 1 USD ≈ 1 EUR).

1.5 Estimate of RoboEM performance for soma-based iterative axon reconstruction in mm^3-scale datasets

In the mouse cortex multiSEM dataset, we performed a simulated soma-based iterative axon reconstruction to estimate expected reconstructed axon path length when combining state-of-the-art agglomeration with iterative RoboEM-based split correction. To this end the branching data from 10 manually reconstructed axons randomly sampled from a barrel centered volume of size 250x200x70 µm^3 (five spiny stellate cells, four star pyramids, and one layer 3 pyramidal cell) were used. We assumed a 90% recall for ending detection (Bernoulli process), Poisson-distributed split errors with an inter split distance of 20 µm for the agglomeration (52 splits/mm

without length threshold as measured in Si150L4 for dense seeded axons) and 17 μm for RoboEM (60 errors/mm reset-based error rate as measured in Si150L4 for dense seeded axons; factor of 2 for validation tracing in backwards direction already taken into account) and applied a minimum agglomerate length threshold of 2.5 μm. Per split the minimum error-free RoboEM distance required to be solved to attach to the next agglomerate was set to 0.5 μm. Under this model, the reconstructed path length attached to the soma can be increased from 35 ± 15 μm to 2.0 ± 0.5 mm using RoboEM (N=10 Monte Carlo runs). When considering the higher reconstruction accuracy for soma-proximal axons (Extended Data Fig. 3) by assuming ~1 mm and 2 mm of path length already attached to the soma, RoboEM split correction yields 4.7 ± 0.3 mm and 6.0 ± 0.3 mm of reconstructed axon path length, respectively (N=10 Monte Carlo runs).

2 SUPPLEMENTARY FIGURE

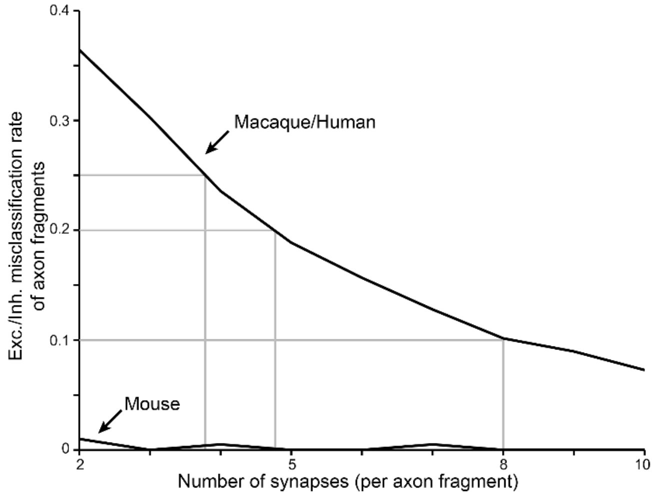

Supplementary Figure 1. Minimal required axon lengths for connectomic analyses. Dependence of excitatory/inhibitory axon classification on length of faithfully reconstructed axons (reported as number of synapses per axon fragment). Misclassification rates for cortical axon fragments versus number of synapses per fragment shown, based on model and data from (Loomba et al., 2022). While in mouse cortex the distinction of excitatory versus inhibitory can already be made with high accuracy based on 2-3 synapses, in macaque and human cortex 4 and 8 synapses are required to have a misclassification rate of around 25% and 10% respectively.

3 SUPPLEMENTARY TABLES

Tracing considered	Distance Γ to ground truth	Angle θ to ground truth
**correct**	$\Gamma \leq 360$ nm	$\theta \leq 90°$
**experimental** → progress along ground truth not considered	360 nm < $\Gamma \leq$ 800 nm	$90° < \theta \leq 160°$
**wrong** → reset to ground truth	$\Gamma > 800$ nm	$\theta > 160°$

Suppl. Table 1: Thresholds employed for categorization of the tracing state allowing for reset-based error rate determination on validation sets.

	(I) autoAggl.	(II) autoAggl. + Human corrections	(III) autoAggl. + RoboEM corrections
# axons ≥10 synapses	2623	6979	6795
# excitatory axons ≥10 synapses	1904	5894	5599
# inhibitory axons ≥10 synapses	651	893	1058
# synapses onto soma	4668	4742	5101
# synapses onto whole cells	33164	45706	43923
# synapses onto apical dendrites	9816	14090	12985
# synapses onto smooth dendrite	15397	17908	18554
# synapses onto AIS	547	615	689
# synapses onto other	96230	149431	138910

Suppl. Table 2: Quantitative comparison of reconstruction results in the mouse cortex SBEM dataset, see Extended Data Fig. 2.

<table>
<tr><td rowspan="2">Dataset, base reconstruction pipeline
(RoboEM-based error correction applied in all cases)</td><td>Minor merge errors</td><td colspan="2">Major merge errors</td></tr>
<tr><td>≤ 1μm overlap with ground truth</td><td>3D-EM data related (misalignment, artifacts, missing sections)</td><td>Other causes</td></tr>
<tr><td rowspan="2">multiSEM, FFN c2, Human Cortex
(Shapson-Coe et al., 2021)
1.43 mm axon length</td><td rowspan="2">14% (1/7)
(caused by RoboEM: 0)</td><td colspan="2">86% (6/7)</td></tr>
<tr><td>83% (5/6)
(caused by RoboEM: 0)</td><td>17% (1/6)
(caused by RoboEM: 1)</td></tr>
<tr><td rowspan="2">multiSEM, autoAggl., Mouse cortex
(Si150L4)
1.66 mm axon length</td><td rowspan="2">77% (10/13)
(caused by RoboEM: 0)</td><td colspan="2">23% (3/13)</td></tr>
<tr><td>100% (3/3)
caused by RoboEM: 1)</td><td>0% (0/3)</td></tr>
</table>

Suppl. Table 3: Evaluation of merge errors on dense axons after RoboEM correction in the multiSEM datasets, see Figure 2.

Approach	Hardware	Run-time	CPU core hours	GPU node hours	Dataset size factor	Cost [EUR/m]
**FFN** (Januszewski et al., 2018)	1000x NVIDIA Tesla P100 nodes *(2.992 USD/h/P100)*	6.964 h	-	6964	0.5	3904
**Dense reconstruction** (Motta et al., 2019)	24x CPU nodes with 16 CPU cores each @ 16 GB RAM / core *(0.0835 USD/h/core)*	101 h	38.8 k		1	1200
**LSD** (here: AcRLSD) (Sheridan et al., 2022)	24x NVIDIA Tesla V100 nodes for CNN inference *(3.808 USD/h/V100)*	10.5 h	-	252		
	100 CPU cores for watershed and agglomeration *(0.0835 USD/h/core)*	7.7 h	0.77 k	-	0.7	266
**RoboEM correction**	1x NVIDIA Tesla V100 node with 32 CPU cores @ 128GB RAM *(3.808 USD/h/V100)*	35 h	-	35	1	49

Suppl. Table 4: Comparison of computational costs for various reconstruction pipelines, see Fig. 1i.

4 REFERENCES

Januszewski, M., Kornfeld, J., Li, P. H., Pope, A., Blakely, T., Lindsey, L., . . . Jain, V. (2018). High-precision automated reconstruction of neurons with flood-filling networks. *Nature Methods*. doi:10.1038/s41592-018-0049-4

Loomba, S., Straehle, J., Gangadharan, V., Heike, N., Khalifa, A., Motta, A., . . . Helmstaedter, M. (2022). Connectomic comparison of mouse and human cortex. *Science*. doi:10.1126/SCIENCE.ABO0924

Motta, A., Berning, M., Boergens, K. M., Staffler, B., Beining, M., Loomba, S., . . . Helmstaedter, M. (2019). Dense connectomic reconstruction in layer 4 of the somatosensory cortex. *Science*, eaay3134. doi:10.1126/science.aay3134

Shapson-Coe, A., Januszewski, M., Berger, D. R., Pope, A., Wu, Y., Blakely, T., . . . Lichtman, J. W. (2021). A connectomic study of a petascale fragment of human cerebral cortex. *bioRxiv*, 2021.2005.2029.446289. doi:10.1101/2021.05.29.446289

Sheridan, A., Nguyen, T. M., Deb, D., Lee, W. C. A., Saalfeld, S., Turaga, S. C., . . . Funke, J. (2022). Local shape descriptors for neuron segmentation. *Nature Methods, 20*, 295-303. doi:10.1038/s41592-022-01711-z
