# Supplementary material for: RoboEM: automated 3D flight tracing for synaptic-resolution connectomics
Source: Nat Methods. 2024 Mar 21;21(5):908–13. doi: 10.1038/s41592-024-02226-5 (PMC11093750; doi:10.1038/s41592-024-02226-5)
Supplement: Supplementary file 2 — Reporting Summary [file 41592_2024_2226_MOESM2_ESM.pdf]

Corresponding author(s): Helmstaedter, Moritz

Last updated by author(s): Nov 23, 2023

Reporting Summary

Nature Portfolio wishes to improve the reproducibility of the work that we publish. This form provides structure for consistency and transparency in reporting. For further information on Nature Portfolio policies, see our Editorial Policies and the Editorial Policy Checklist.

Statistics

For all statistical analyses, confirm that the following items are present in the figure legend, table legend, main text, or Methods section.

n/a	Confirmed	
☐	☒	The exact sample size (*n*) for each experimental group/condition, given as a discrete number and unit of measurement
☒	☐	A statement on whether measurements were taken from distinct samples or whether the same sample was measured repeatedly
☐	☒	The statistical test(s) used AND whether they are one- or two-sided<br>*Only common tests should be described solely by name; describe more complex techniques in the Methods section.*
☒	☐	A description of all covariates tested
☒	☐	A description of any assumptions or corrections, such as tests of normality and adjustment for multiple comparisons
☐	☒	A full description of the statistical parameters including central tendency (e.g. means) or other basic estimates (e.g. regression coefficient) AND variation (e.g. standard deviation) or associated estimates of uncertainty (e.g. confidence intervals)
☒	☐	For null hypothesis testing, the test statistic (e.g. *F*, *t*, *r*) with confidence intervals, effect sizes, degrees of freedom and *P* value noted<br>*Give P values as exact values whenever suitable.*
☐	☒	For Bayesian analysis, information on the choice of priors and Markov chain Monte Carlo settings
☒	☐	For hierarchical and complex designs, identification of the appropriate level for tests and full reporting of outcomes
☒	☐	Estimates of effect sizes (e.g. Cohen's *d*, Pearson's *r*), indicating how they were calculated

Our web collection on statistics for biologists contains articles on many of the points above.

Software and code

Policy information about availability of computer code

Data collection	No software was used.
Data analysis	All source code and binary files are publicly available in the supplement under the following provisions: Any copyright or patent right is owned by and proprietary material of the Max-Planck-Gesellschaft zur Förderung der Wissenschaften e.V. ("MPG"). MPG makes no representations or warranties of any kind concerning the source code and binary files, neither express nor implied, and the absence of any legal or actual defects, whether discoverable or not.<br>The source code and binary files available in the supplement are subject to a non-exclusive, revocable, non-transferable, and limited right to use for the exclusive purpose of undertaking academic or not-for-profit research. Use of the Code or binary files or any part thereof for commercial or clinical purposes and alterations are strictly prohibited in the absence of a Commercial License Agreement from MPG. Download and/or use of the source code and binary files under the aforementioned research license comes with the acceptance of the license agreement provided with the download. Any further distribution is prohibited.<br><br>Publicly available software used in this study:<br>* Segmentation software SegEM (https://github.com/mhlabCodingTeam/SegEM/, version: 1.0) from Berning et al. Neuron (2015)<br>* Synapse detection software SynEM (https://gitlab.mpcdf.mpg.de/connectomics/SynEM) from Staffler et al. eLife (2017)<br>* Analysis algorithms (https://gitlab.mpcdf.mpg.de/connectomics/L4dense/) from Motta et al. Science (2019)<br>* Skeletonization software Kimimaro (https://github.com/seung-lab/kimimaro, version: 0.2.1) from Silversmith et al. (2021)<br>* Deep learning software Tensorflow (https://www.tensorflow.org/, version: 2.3.0) from Abadi et al. OSDI'16 (2016)

For manuscripts utilizing custom algorithms or software that are central to the research but not yet described in published literature, software must be made available to editors and reviewers. We strongly encourage code deposition in a community repository (e.g. GitHub). See the Nature Portfolio guidelines for submitting code & software for further information.

Data

Policy information about availability of data

All manuscripts must include a data availability statement. This statement should provide the following information, where applicable:

- Accession codes, unique identifiers, or web links for publicly available datasets
- A description of any restrictions on data availability
- For clinical datasets or third party data, please ensure that the statement adheres to our policy

All data necessary to reproduce reported results for the mouse cortex SBEM dataset (Motta et al. 2019) are available: https://wklink.org/9276 (raw data), https://L4dense2019.brain.mpg.de (code and data of the original publication); trained RoboEM weights, and evaluation data are available in the supplement subject to provisions as stated in the code availability section (see above).
Raw data for the mouse cortex multiSEM dataset are available: Si11L3 https://wklink.org/2458 (spine head attachment test set), Si150L4 https://wklink.org/7122 (axon test set); RoboEM training data and trained RoboEM weights to reproduce results for the mouse cortex multiSEM dataset are available upon reasonable request. Raw data for the human cortex multiSEM dataset (Shapson-Coe et al. 2021) are available: https://h01-release.storage.googleapis.com/landing.html; RoboEM training data and trained RoboEM weights to reproduce results for the human cortex multiSEM dataset are available upon reasonable request.
In addition, data needed to run the example code is part of the zipped code package provided in the supplementary material subject to provisions as stated in the code availability section (see above).

Human research participants

Policy information about studies involving human research participants and Sex and Gender in Research.

Reporting on sex and gender	n/a
Population characteristics	n/a
Recruitment	n/a
Ethics oversight	n/a

Note that full information on the approval of the study protocol must also be provided in the manuscript.

Field-specific reporting

Please select the one below that is the best fit for your research. If you are not sure, read the appropriate sections before making your selection.

☒ Life sciences ☐ Behavioural & social sciences ☐ Ecological, evolutionary & environmental sciences

For a reference copy of the document with all sections, see nature.com/documents/nr-reporting-summary-flat.pdf

Life sciences study design

All studies must disclose on these points even when the disclosure is negative.

Sample size	n=3 three dimensional electron microscopy datasets were used to ensure variabilities from acquision/imaging modalities (SBEM versus multiSEM) and species (mouse versus human) have no negative impact on performance; however, the main conclusions of this study rely on the sample sizes and/or path lengths from manually proof-read axons (see below);<br>n=90 ending queries and n=100 chiasma queries of axons were used to compare RoboEM neurite tracings with human neurite tracings;<br>n=5 random axons for the two multiSEM datasets and n=10 random axons for the SBEM dataset were used with similar total path lengths of over 1 millimeter were used for evaluation of split and merge error rates;<br>n=91 spine heads were used in the multiSEM dataset by Sievers et al. to evaluate attachment of spine heads using RoboEM
Data exclusions	No data were excluded.
Replication	Reconstruction algorithms as described in this study were successfully run on multiple datasets by different scientists confirming the results of this study.<br>Source code and data required to reproduce results are made available as indicated in the code and data availability sections.
Randomization	All samples were selected randomly without input from investigators.<br>Randomization was done using pseudo-random number generators in MATLAB or Python.
Blinding	N/A

Reporting for specific materials, systems and methods

We require information from authors about some types of materials, experimental systems and methods used in many studies. Here, indicate whether each material, system or method listed is relevant to your study. If you are not sure if a list item applies to your research, read the appropriate section before selecting a response.

Materials & experimental systems

n/a	Involved in the study	
☒	☐	Antibodies
☒	☐	Eukaryotic cell lines
☒	☐	Palaeontology and archaeology
☐	☒	Animals and other organisms
☒	☐	Clinical data
☒	☐	Dual use research of concern

Methods

n/a	Involved in the study	
☒	☐	ChIP-seq
☒	☐	Flow cytometry
☒	☐	MRI-based neuroimaging

Animals and other research organisms

Policy information about studies involving animals; ARRIVE guidelines recommended for reporting animal research, and Sex and Gender in Research

Laboratory animals	Experiments were performed on laboratory animals, species mus musculus, strain C57BL/6-J from age P5 until 6 months.
Wild animals	No wild animals were used.
Reporting on sex	Experiments were approved for male mice. Sex was approved in animal husbandry and only male mice were used for experiments and sample preparation.<br>Reason for exclusion of females was comparability with already published data, no further knowledge so far about the influence of estrus, combined with low sample numbers prepared in the studies.
Field-collected samples	No field collected samples were generated/used.
Ethics oversight	Experiments were performed as part of the approved animal research project “Nervenzellschaltkreise Grosshirnrinde Nager”. Approval was granted from the Animal Welfare Agency, Regional Council of Darmstadt/Tierschutzbehörde, Regierungspräsidium Darmstadt, file number: V54-19c20/15-F126/1002. The approval was granted from Feb. 18th 2015 until Feb 17th 2020.<br>Ethical guidance on the study protocol was provided by the Animal Welfare Officer of the Max Planck Institute for Brain Research.

Note that full information on the approval of the study protocol must also be provided in the manuscript.
